# Integrated Analysis of Long Non-Coding RNA and mRNA to Reveal Putative Candidate Genes Associated with Backfat Quality in Beijing Black Pig

**DOI:** 10.3390/foods11223654

**Published:** 2022-11-15

**Authors:** Xin Liu, Weilong Tian, Ligang Wang, Longchao Zhang, Jing Liang, Lixian Wang

**Affiliations:** 1 Institute of Animal Sciences, Chinese Academy of Agricultural Sciences, Beijing 100193, China; 2 College of Animal Science and Technology, Guangxi University, Nanning 530004, China

**Keywords:** pigs, backfat quality, mRNAs, lncRNAs, candidate genes

## Abstract

Pigs’ backfat quality has an important impact on the quality of pork and pork products and has a strong relationship with nutrition and sensory characteristics. This study aimed to identify the related candidate genes of backfat quality and to preliminary clarify the molecular regulatory mechanism underlying pig backfat quality phenotypes. Expression assessments of long non-coding RNA (lncRNA) and mRNA profiling in backfat from high-quality (firm and white) and low-quality (soft and yellow) Beijing Black pigs were performed by RNA sequencing. Significantly different expressions were observed in 610 protein-coding genes and 290 lncRNAs between the two groups. Gene Ontology and Kyoto Encyclopedia of Genes and Genomes pathway annotation showed that some candidate differentially expressed genes that participate in lipid-related pathways and pigmentation terms may play a role in backfat quality in pigs. The cis-target and trans-target genes were predicted to explore the regulatory function of lncRNAs, and integrative analyses of different expression lncRNAs targets and different expression genes were performed. The results showed the regulatory networks of lncRNA-mRNA related to backfat quality, and our study obtained strong candidate genes for backfat quality: *ELOVL5*, *SCD*, *DGAT2*, *SLC24A5,* and *TYRP1*, which were involved in fat metabolism, adipogenesis regulation, and pigmentation. To our knowledge, this study is the first to demonstrate the molecular genetic mechanisms of backfat quality in pigs, and these findings improve the current understanding of backfat quality mechanisms and provide a foundation for further studies.

## 1. Introduction

Fat is an important characteristic determining the quality of pork. Fat quality is considered a significant factor that has an important impact on the quality of pork and pork products and even determines the overall value of the carcass, given its strong relationship with nutrition, shelf life, safety, and sensory characteristics [1]. Good-quality backfat is defined as firm and white, and poor fat is defined as soft, oily, wet, gray, and floppy [2]. As a visible feature of pork, the fat color will directly affect consumers’ judgment of meat quality and their purchase decisions. Consumers prefer white fat in pork carcasses and are less likely to accept yellow fat. Moreover, the color of fat will turn yellow with long storage times [3]. The yellow appearance of fat is caused by carotenoid pigments within adipocytes or diseases [1]. Fat firmness is another important parameter of fat quality. Soft fats cause reductions in the quality of cuts and lead to losses of value as they are difficult to dimension and process; they are also not generally accepted by consumers for their oily appearance [1,4]. Firm fat is the ideal quality parameter required when producing bacon and sausage [5]. As such, fat quality, comprising fat color and firmness, has now been assigned importance by many pork enterprises and processing industries.

It is generally believed that proteins carry more genetic information. However, based on the rapid development of next-generation sequencing technologies, high-throughput RNA-seq has become widely used for estimating transcriptome profiles, and recent studies have shown that gene expression is also regulated by non-coding RNAs (ncRNAs) [6,7,8]. The long non-coding RNA (lncRNA), as a type of ncRNA transcript, is longer than 200 nt in length and has almost no protein-coding ability [9,10]. As such, many lncRNAs have been recognized by high-through RNA-sequencing technology, and recent studies have shown lncRNAs play a key role in regulating gene expression at the transcriptional and post-transcriptional level, enabling them to perform their biological function in mammalian physiological and pathological processes via their unique sequence and structure [9,11,12]. Additionally, some candidate lncRNAs and lncRNA-mRNA pathways have been defined, and the regulatory effects on some biosynthesis, metabolism, and disease processes have been validated [13,14,15,16]. Backfat deposition is a complex biological process regulated by multiple genes and epigenetic factors, including lncRNAs [17]. There have been some reports on lnRNAs related to fat deposition and metabolism in various species [18,19,20]; however, the lncRNAs related to fat color and firmness, and their regulatory relation profiles with the corresponding mRNA, remain unclear.

The Beijing Black pig is a black pig breed cultivated in China and is best known for its favorable characteristics, including the superior quality of the meat it yields [21]. In this study, our study used high-through RNA-seq technology to systematically identify the lncRNAs and mRNAs of the backfat tissue from high-quality (firm and white) and low-quality (soft and yellow) Beijing Black pigs, and differential expression and integrated analysis were used to assess the molecular mechanisms underlying the backfat firmness of pigs. This study identified key candidate genes and lncRNAs for the backfat quality of pigs, and it provides a new reference for further research on the genetic mechanism of pig backfat quality.

## 2. Materials and Methods

### 2.1. Ethics Statement

The study was approved by the Ethics Committee of the Institute of Animal Sciences of the Chinese Academy of Agricultural Sciences (IAS2022-38). All experimental protocols were conducted in accordance with the approved guidelines.

### 2.2. Animals and Sample Collection

The 400 female Beijing black pigs used in this experiment were provided by Beijing Heiliu Animal Husbandry Technology Co., Ltd. (Beijing, China), and all pigs were raised under the same environmental and nutritional conditions. The 240 ± 7 days pigs were weighed and sacrificed in a commercial abattoir. Backfat was collected within 2 h after slaughter from the carcass’s left side. The skin was removed, and fat samples were collected in coded plastic bags. The samples were placed in a 4 °C refrigerator for subsequent fat color and firmness analysis. At the same time, the same parts of the fresh backfat tissues were used for RNA extraction. Then, they were immediately frozen in liquid nitrogen, followed by storage in a refrigerator at −80 °C until use.

### 2.3. Phenotypic Traits of Backfat Analysis

The color of the backfat sample was measured at 24 h after sacrifice. The values of CIELAB L, a, and b were tested by using a Minolta CR200 instrument (Osaka, Japan) according to the standard instrumental procedure. L represents the surface lightness/darkness, a represents redness/greenness, and b represents yellowness/blueness. The b value was recognized as an index for evaluating the yellowness of fat surface, and therefore, the b value was the main focus of this study [22].

The backfat firmness was measured via the methods of a previous study [3]. The backfat firmness samples were measured by a TA-XT2 texture analyzer (Stable Micro Systems Ltd., Surrey, UK) with a flat-ended cylindrical probe (SMS P/0.5). The backfat sample with an average temperature of 7 °C was placed on the platform, and the outer layer was placed downwards. Each sample was trimmed to 1 cm^3,^ and five duplicate samples were taken from every pig. The parameters of the firmness test were a speed of 1.0 mm/s and compression until 90% thickness. Peak force (Newton, N) used as a measure of firmness was recorded. The average value of five measurements was taken as the backfat firmness of the sample. 

According to the population phenotypic data, three individuals with lower b values and higher firmness of backfat were selected as the high group (BH), and three individuals with higher b values and lower firmness were selected as the low group (BL). The phenotypic values of the two groups were significantly different, and there were >2 standard deviations (Table 1).

### 2.4. RNA Extraction and Qualification

The total RNA of backfat tissue was isolated using TRIzol reagent (Invitrogen, Carlsbad, CA, USA). RNA degradation and contamination were detected by 1% agarose gel electrophoresis. A nanodrop spectrophotometer (Nanodrop Technologies, Wilmington, DE, USA) was used to determine RNA purity and concentration. RNA integrity and quantity were measured using a Bioanalyzer 2100 (Agilent Technologies, Santa Clara, CA, USA).

### 2.5. Library Preparation and Sequencing

After performing quality control (QC), the samples were used for library preparation. RNA-seq libraries were prepared with approximately 3 μg total RNA from each sample. After removing ribosomal RNAs (rRNAs), libraries for strand-specific RNA sequencing were constructed. RNA was segmented by RNase R enzyme and then used as a template to synthesize cDNA. After the degradedness of RNA, the second cDNA strand was synthesized by using the DNA polymerase I system. The library fragments were ligated to NEBNext Adaptor and purified with the AMPure XP system (Beckman Coulter, Beverly, MA, USA). Finally, the amplification of the library by polymerase chain reaction (PCR) was performed. The six cDNA libraries were sequenced on an Illumina HiSeq 2500 platform (Illumina, San Diego, CA, USA). All of the clean data generated from this study have been submitted to the Genome Sequence Archive, with the accession number CRA007928.

### 2.6. Read Mapping and Transcriptome Assembly

First, raw reads, which were in FASTQ format, were evaluated and filtered by fastp (V0.18.0) [23] and Trimmomatic (version 0.36) [24] to obtain high clean reads. The high-quality clean reads were mapped to the pig reference genome (*Sus scrofa* 11.1, http://ftp.ensembl.org/pub/release-105/fasta/sus_scrofa/dna/ (accessed on 1 March 2022)) using HISAT2 version 2.1.0 with the default parameters [25]. Assemble the mapped reads of each library with StringTie (V1.3.4) with default parameters [26]. Then, our study used StringTie to merge the six assembled transcript files (GTF format) of three groups into a nonredundant transcriptome.

### 2.7. Identification of Potential Candidate lncRNAs

The lncRNAs were identified according to the following strict filtering criteria: (1) Our study removed transcripts overlapping with pig protein-coding genes. (2) Our study removed transcripts shorter than 200 nt, with an exon number less than two. (3) Our study removed low-expressed transcripts with FPKM < 0.5. (4) Our study removed transcripts overlapping with other non-coding RNAs, such as tRNA, rRNA, snRNA, and snoRNA). (5) Our study removed the transcripts located within annotated genes’ 1kb flanking regions. (6) Transcripts without coding potential, as predicted by the Coding-Non-CodingIndex (v2) [27] and Coding Potential Calculator (v0.9-r2) [28] database, were novel lncRNAs.

### 2.8. Identification of Differentially Expressed lncRNA and mRNA

lncRNA and mRNA expression levels were calculated and normalized using the fragment per kilobase of transcript per million mapped reads (FPKM). R package DESeq2 [29] was used to analyze the significance of the differentially expressed mRNAs (DEGs) and lncRNAs (DELs) between BH and BL. In the screening results, mRNAs and lncRNAs with adjusted-*p* values of <0.05 and absolute FoldChange values of >1.5 were considered to be DEGs and DELs.

### 2.9. Target Gene Prediction of DELs

To determine the relevant functions of lncRNAs, target gene predictions are first needed. At present, the main prediction methods are cis-regulating nearby protein-coding genes and trans-regulating distant protein-coding genes. The target genes of DELs were analyzed as follows: Bedtools was used to search for protein-coding genes 100 kb upstream or downstream of each putative DEL, which were considered potential cis-target genes (CTGs) of DELs [30]. The Pearson correlation coefficient (PCC) of the expression values of DELs and mRNA was calculated, and the genes with |PCC| > 0.99 and *p* < 0.05 were selected as the trans-target genes (TTGs) of DELs.

### 2.10. Functional Enrichment of DEGs and Target Genes of DELs

The DEGs, CTGs, and TTGs performed enrichment analysis of Gene ontology (GO) analysis and Kyoto Encyclopedia of Genes and Genomes (KEGG) pathway by clusterProfiler package [31]. GO terms or KEGG pathways with *p* < 0.05 were significant. KEGG pathways related to fat deposition and metabolism were visualized using Cytoscape software [32].

An expression–regulation network was constructed by mRNAs and lncRNAs related to lipid deposition and metabolism. According to the results of the correlation coefficient analysis as well as KEGG analyses, the DEGs related to fat metabolism were selected (|PCC| > 0.9) for co-expression construction to identify the key lncRNAs regulating fat deposition.

### 2.11. Quantitative Real-Time PCR Validation

In order to validate the reliability of RNA-seq data in Beijing Black pig, five DEGs (*ME1*, *SCD*, *PPARG*, *ACLY,* and *ACE2*) and six DELs (TCONS_00012871, TCONS_00224182, TCONS_00250121, TCONS_00208232, TCONS_00148627, and TCONS_00282800) were randomly selected for qRT-PCR. The total RNA of the fat sample was extracted using Trizol reagent (Invitrogen, Carlsbad, CA, USA), according to the manufacturer’s instructions, and then reverse transcribed into cDNA with the Revert Aid First Strand cDNA Synthesis Kit (Thermo Scientific, Waltham, MA, USA). The qRT-PCR amplification was performed on ABI 7900HT (Applied Biosystems, Foster City, CA, USA). The *GAPGH* gene was tested as an internal control. The primers of mRNAs and LncRNAs are shown in Table S1. The relative expression level of each validated gene was calculated by the 2^−ΔΔCt^ method.

## 3. Results

### 3.1. Overview of Sequencing Data

A total of six cDNA libraries (BH_1, BH_2, BH_3, BL_1, BL_2, BL_3) were constructed for the subcutaneous adipose tissue of Beijing Black pigs, and each was sequenced using Illumina HiSeq 2500 platform. After quality control for raw sequencing data, an average of 92236919 and 93422397 clean reads were collected, respectively. The Q20 of each sample was over 96.36%, and the Q30 was over 90.73%. The GC contents of all samples ranged from 56.87% to 62.37% (Table S2). Approximately 89.81% to 92.89% of total clean reads were mapped to the *Sus scrofa* reference genome (Sscrofa11.1), and 67.75% to 77.78% of the reads were uniquely mapped to specific regions of the porcine reference genome, respectively (Table S3). These results indicate that the sequencing data were reliable and could be used for subsequent bioinformatics analysis.

### 3.2. Identification and Characteristics of lncRNAs and mRNAs

**Figure 1:**
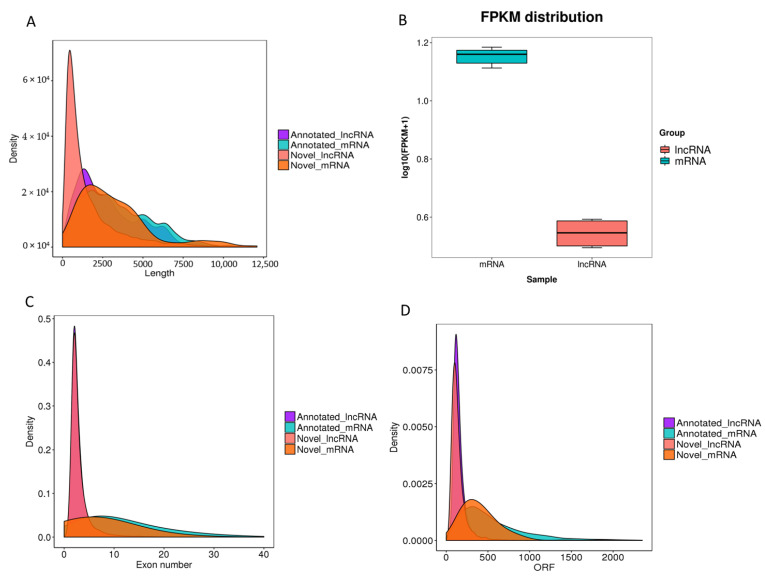
Genomic characteristics of mRNAs and lncRNAs in pig backfat (**A**) Length distribution of annotated mRNAs, annotated lncRNAs, novel mRNAs, and novel lncRNAs; (**B**) FPKM value of mRNAs and lncRNAs; (**C**) Exon number distribution of annotated mRNAs, annotated lncRNAs, novel mRNAs and novel lncRNAs; (**D**) ORF length distribution of annotated mRNAs, annotated lncRNAs, novel mRNAs and novel lncRNAs.

Based on the transcriptome, Our study obtained 39123 protein-coding transcripts (38982 known protein-coding transcripts and 141 novel protein-coding transcripts) and 13979 lncRNA transcripts (6646 known lncRNA transcripts and 7333 novel lncRNA transcripts). The average transcript lengths and their expressions of lncRNAs were less than those of protein-coding transcripts (Figure 1A,B). Meanwhile, the average exon numbers of protein-coding transcripts were much larger than those of lncRNAs (Figure 1C). Furthermore, our results showed that known and novel lncRNAs had shorter open reading frame (ORF) lengths compared with those of mRNA transcripts (Figure 1D). The sub-figures of Figure 1A,C,D are shown in Figure S1.

### 3.3. Analysis of Differentially Expressed mRNAs and lncRNAs

**Figure 2:**
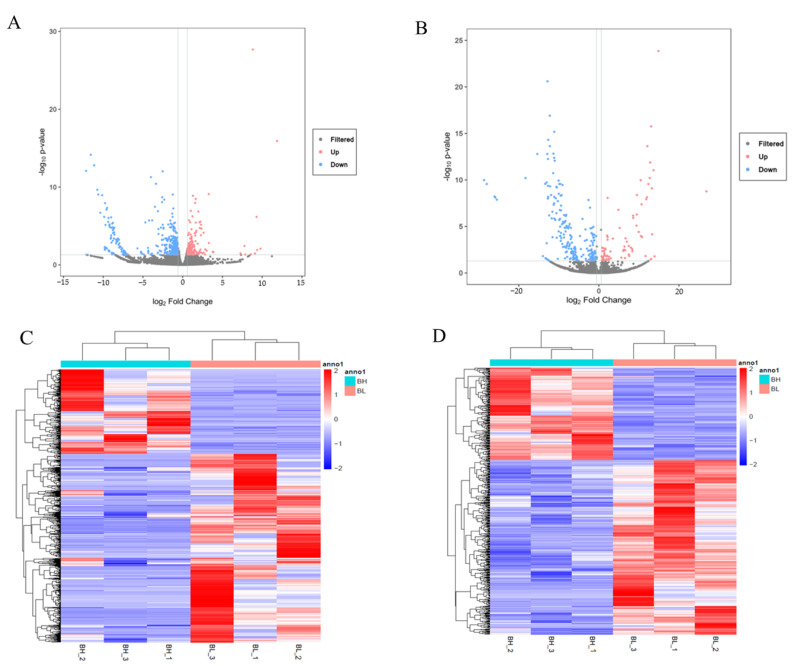
Expression profiles of distinct RNAs in backfat. (**A**) Volcano plots of DEGs; (**B**) Volcano plots of DELs; (**C**) Heatmap of DEGs; (**D**) Heatmap of DELs.

To identify mRNAs and lncRNAs related to differences in backfat quality between BH and BL Beijing Black pigs, DEGs and DELs were performed after the basic analysis of sequencing results. A total of 610 DEGs (Table S4), including 212 upregulated genes and 398 downregulated genes, and 290 DELs (Table S5), including 77 upregulated lncRNAs and 213 downregulated lncRNAs, were finally identified. The volcano plots (Figure 2A,B) and heatmaps (Figure 2C,D) show the clusters of the DEGs and DELs based on the expression. The results show similar expression patterns and relationships in each group. 

### 3.4. GO Terms and KEGGPathways Analysis of DEGs

**Figure 3:**
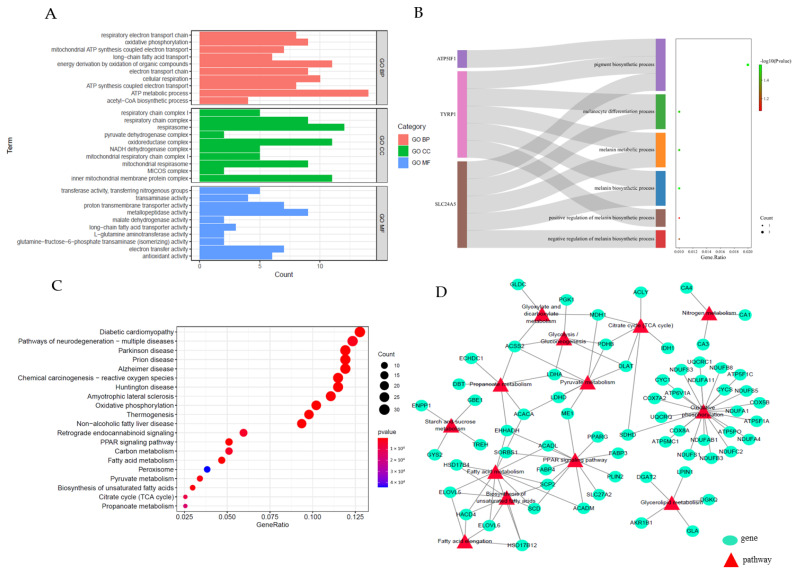
Functional enrichment analyses of DEGs: (**A**) GO enrichment annotation of DEGs; (**B**) Significant GO-BP terms related to pigmentation; (**C**) KEGG enrichment annotation of DEGs; (**D**) The interaction network between lipid-related mRNAs-pathway.

In order to reveal the potential function of DEGs between the BH and BL of Beijing Black pigs, GO and KEGG enrichment analyses were applied to the DEGs. A total of 361 GO terms were significantly enriched (*p* < 0.05). Our study identified the top 10 significantly enriched GO terms of the biological process, including the ATP metabolic process, the electron transport chain, and long-chain fatty acid transport (Figure 3A). In particular, our study found that six terms were related to pigmentation in GO-BP, and the *SLC24A5* and *TYRP1* genes were simultaneously enriched in several items related to pigmentation (Figure 3B). The top 20 most significant pathways (*p* < 0.05) were enriched by DEGs, as shown in Figure 3C, including some lipid-related pathways: PPAR signaling pathway, fatty acid metabolism, biosynthesis of unsaturated fatty acids, and so on. Our study constructed a lipid-related mRNAs–pathway co-expression network, and our study found *ACACA*, *ELOVL5*, *ME1*, *ELOVL6*, *DGAT2,* and *SCD* genes which functioned in fat synthesis, deposition, and metabolism and enriched in multiple lipid metabolism-related pathways (Figure 3D). This implies that these pathways and related genes may play a key role in regulating the backfat quality of Beijing Black pigs.

### 3.5. Identification and Function Enrichment of Target Genes of DELs

**Figure 4:**
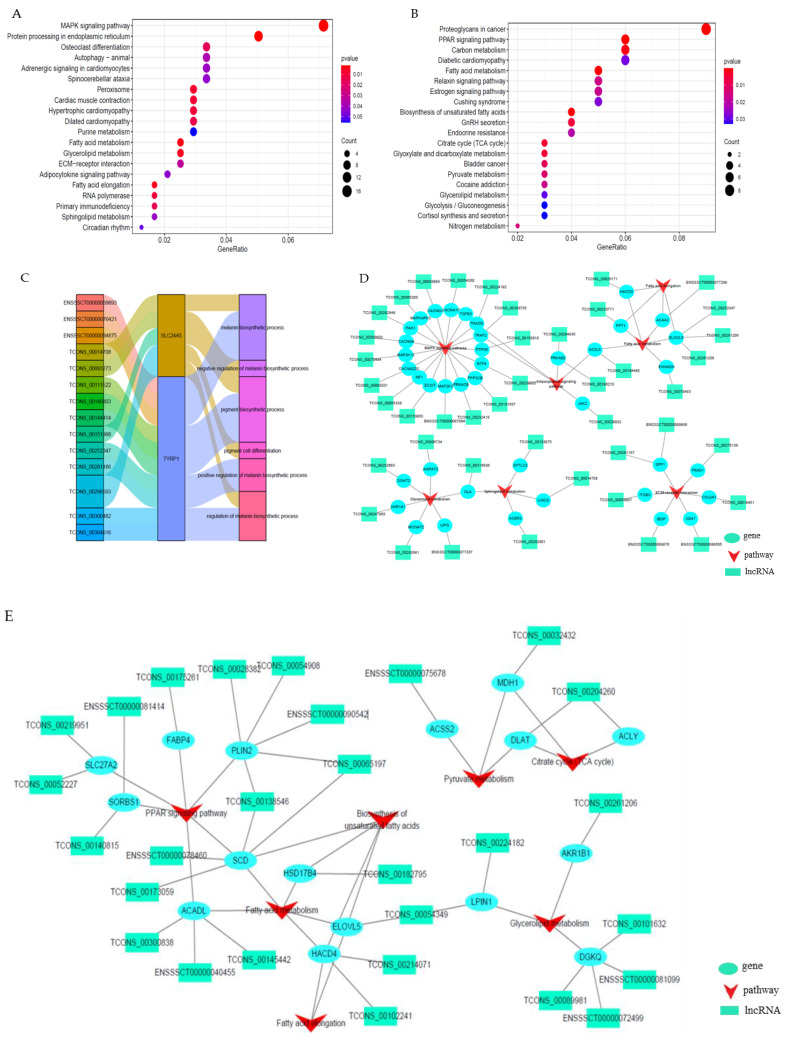
Functional enrichment analyses of DELs: (**A**) KEGG pathways enrichment annotation of CTGs; (**B**) KEGG pathways enrichment annotation of GTGs; (**C**) Significant GO-BP terms related to pigmentation; (**D**) The interaction network between DELs-CTGs-pathway; (**E**) The interaction network between DELs-TTGs-pathway.

To further analyze the potential function of DELs in regulating the backfat quality of Beijing Black pigs, the potential CTGs and TTGs were identified first, and then GO and KEGG enrichment analyses were carried out to investigate the roles of lncRNAs in CTGs and TTGs. The results showed that 984 CTGs and 297 TTGs were obtained (Tables S6 and S7). CTGs were involved in the biological processes, including the regulation of the sequestering of triglyceride, the lipoprotein metabolic process, the regulation of lipid transport, the MAPK signaling pathway, and the fatty acid-related metabolism (Figure 4A). The TGGs were significantly enriched in some processes and pathways related to lipids, such as lipid transport, lipid metabolic process, fatty acid metabolism, the PPAR signaling pathway, the biosynthesis of unsaturated fatty acids, and so on (Figure 4B). In particular, two genes, *SLC24A5* and *TYRP1,* were found to be significantly enriched in the six terms related to pigmentation, and this was consistent with those obtained by DEGs analysis. As such, our study hypothesizes that *SLC24A5* and *TYRP1* genes could be the key genes, and they regulate the color of backfat through the lncRNA regulation pathway (Figure 4C). Then, co-expression networks related to the lipid regulation and the metabolism of the DELs–CTGs and DELs–TTGs pathways were constructed (Figure 4D,E). From the networks, our study found that some genes, such as *ELOVL5*, *SCD*, *and DGAT2*, were involved in multiple fat-related pathways and have been reported as related to lipids. So our study inferred they might regulate the quality of backfat. 

### 3.6. LncRNA–mRNA Network Construction

**Figure 5:**
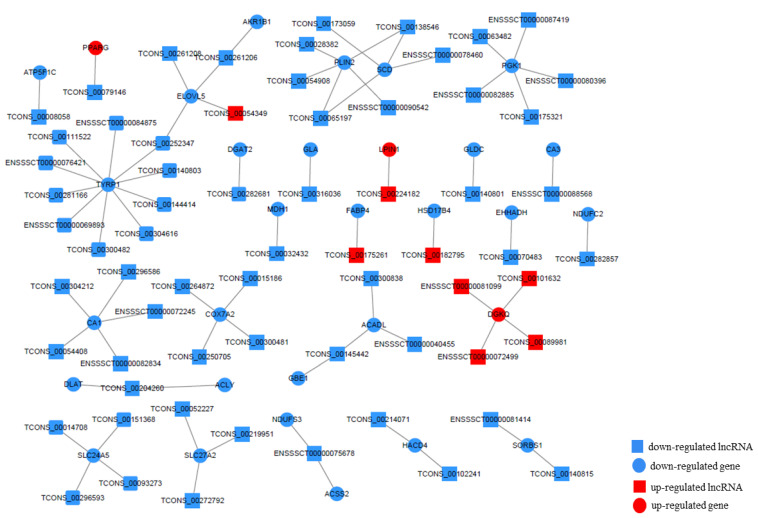
Expression regulation network constructed by differentially expressed mRNA-lnRNA.

To better demonstrate the interaction between DEGs and DELs, the construction of the lnRNA–mRNA network was undertaken (Figure 5). All predicted target genes of DELs were intersected with the identified DEGs related to pigmentation and lipids. The results show that 31 target genes of 66 DELs were found to coincide with the target DEGs. Combining the enrichment analysis and the biological functions of 31 target genes, *SCD*, *ELOVL5*, *DGAT2*, *SLC24A5,* and *TYRP1,* which are involved in fat metabolism, adipogenesis regulation, and pigmentation, were considered to be the key candidate genes for the fat quality of pigs; these were also regulated by some lncRNAs.

### 3.7. Validation of Expression Levels of lncRNA and mRNA Detected in RNA-Seq

**Figure 6:**
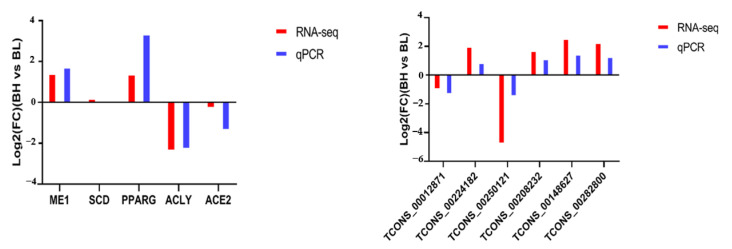
Validation of differentially expressed genes and lncRNAs using qPCR.

To validate the expression levels detected by RNA-Seq, our study randomly selected six lncRNAs (TCONS_00012871, TCONS_00224182, TCONS_00250121, TCONS_00208232, TCONS_00148627, and TCONS_00282800) and five mRNAs (*ME1*, *SCD*, *PPARG*, *ACLY,* and *ACE2*) to conduct qRT-PCR detection. The results show that the expressions of randomly selected lncRNAs and mRNAs were consistent with the results obtained by sequencing (Figure 6). The results demonstrate that the raw RNA-seq data exhibit high reliability and accuracy.

## 4. Discussion

Different species, according to their own characteristics, focus on different parts of fat. Studies on the mechanism of fat in cattle pay more attention to intramuscular fat [33], sheep pay more attention to the characteristics of tail fat [34], and chickens pay more attention to abdominal fat and intramuscular fat [35]. However, in addition to intramuscular fat for pigs, backfat deposition also has an important effect on growth and meat quality, so it is widely studied.

Since increasing growth rate and leanness have been the major objectives of pig breeding programs in recent years, fat accretion has been reduced, and fat content has also changed, leading to negative impacts on the processing aptitude and sensory properties of meat products [36]. With the demand of consumers for high-quality meat and meat products, the fat quality, which is important for both the fresh meat market and high-grade products, has been paid more attention by the pork industry [37]. Backfat deposition and fat traits, considered to be amongst the most essential characteristic of pigs, show strong relationships with the nutritional value of pig products and the technological characteristics of high-quality dry-cured hams [38]. Therefore, backfat deposition affects not only backfat thickness but also fat quality. Backfat deposition is a complex biological process regulated by multiple genes and epigenetic factors, including lncRNA [17]. The genetic mechanism of meat quality-related traits has always been a popular area of research, but the molecular mechanism of fat quality has rarely been reported. RNA-seq has been widely applied to analyze gene expression patterns and identify new transcripts useful for elucidating the molecular function and regulation mechanisms in various organisms due to its high detection sensitivity and low noise [39,40]. In this study, to identify candidate genes and illustrate the molecular regulatory mechanisms of backfat quality, the systematic expression profiles of mRNAs and lncRNAs in backfat tissue of Beijing Black pigs with high- and low-fat quality have been compared by RNA-sequencing. 

Through the detection and comparative analysis of transcriptome patterns of backfat tissues between BH and BL pigs, DEGs were obtained. The biological functions of DEGs were elucidated using GO term enrichment and KEGG pathway analysis. Most genes were enriched in general biological processes, while some genes, mainly enriched in fat-related processes, were identified as candidates for regulating adipogenesis and metabolism, such as *ACACA*, *ELOVL5*, *ME1*, *ELOVL6*, *SCD*, *DGAT2*, *SLC24A5,* and *TYRP1*. *ACACA* is involved in adipose tissue development and lipid metabolism [41], and it encodes acetyl-CoA carboxylase-α, which is a key regulatory enzyme of fatty acid synthesis, catalyzing the carboxylation of acetyl-CoA to malonyl-CoA, leading to the biosynthesis of long-chain fatty acids [42]. The *ACACA* gene was also associated with the fatty acids profile, and synonymous mutations in the *ACACA* gene influenced fatty acid composition in meat, including the polyunsaturated/saturated fatty acid (SFA) ratio [43]. The ELOVL (elongation of very long-chain fatty acids) family members are shown to be mainly involved in mammalian long-chain fatty acid synthesis and are all rate-limiting enzymes [44]. ELOVL5 is a member of the very long-chain fatty acid elongation enzyme family and catalyzes the elongation reaction, which plays an important role in the elongation of various PUFA containing 18 and 20 carbons [45,46]. ELOVL6 plays a key role in catalyzing the elongation of long-chain SFA and MUFA with 12 to 18 carbon fatty acids [47]. The polymorphisms of *ELOVL6*, which regulate gene expression, were strongly associated with fatty acid composition, especially with palmitic and palmitoleic acid contents, in pigs, and different expressions of this gene were shown with extreme phenotypic differences in the intra-muscular fatty acid composition of pigs [48,49]. *ME1* and *SCD* were enriched in the PPAR signaling pathway, which is a recognized pathway closely associated with lipid metabolism [50]. Malic enzyme 1 (ME1), as an NADP-dependent lipogenic enzyme, is a part of the tricarboxylate shuttle that releases acetyl-CoA and NADPH from the mitochondria into the cytosol for fatty acid biosynthesis. Its transcript level, which is higher in the subcutaneous region, could reflect the lipogenic activity of adipose tissue [51,52]. The *ME1* gene is mapped in QTL regions for fatness traits, and the polymorphisms of this gene have been demonstrated to be strongly associated with backfat thickness [53]. The *SCD* gene, which is also mapped in QTL regions significantly related to fatty acid composition, is recognized as a key lipogenic enzyme in the biogenesis of endogenous monounsaturated fatty acids (MUFAs); in particular, it can continue to synthesize MUFAs when dietary unsaturated fat is insufficient [54,55]. There have been reports of *SCD* in regulating adipocyte differentiation, fat deposition, and systemic lipid metabolism by *SCD* knockout or editing tests in animal models [56,57]. DGAT2, as a transmembrane protein, functions in lipid metabolism, especially in triacylglycerol (TG) synthesis. *DGAT2* knocked out mice die after birth with a >90% reduction in TGs, which suggests that *DGAT2* may act as the predominant enzyme for TG storage [58,59]. *DGAT2* is also involved in the carotenoid synthesis, which may be important for yellow pigment deposition in yellow mutant rainbow trout [60]. *SLC24A5*, which encodes a putative cation exchanger, is located on the intracellular membrane, likely the melanosome or its precursor. *SLC24A5* was first identified as a pigmentation-related gene, as it is strongly associated with the golden (hypopigmentation) phenotype in zebrafish, and it was also confirmed to play an important role in the processes of melanin biogenesis and skin pigmentation in animals and human models [61,62,63]. Tyrosinase-related protein 1 (TYRP1), as a melanocyte-specific gene product, contributes to melanin synthesis within melanosomes via its specific expression in melanocytes [64]. The mutation of this gene could affect melanin synthesis; for example, the brown pelage in mice and oculocutaneous albinism type 3 in humans [65]. 

LncRNAs can regulate transcriptional processes via signals and can even interact with other proteins to produce a ribonucleoproten complex as scaffolds. Previous studies have reported that many lncRNAs participate in various biological processes, including fat-related processes, and even play a key regulatory role [17,66,67]. However, the lncRNAs associated with backfat quality in pigs have not been studied. In this study, our study obtained 13,979 lncRNA transcripts. Compared to mRNA transcripts, lncRNAs showed shorter ORF lengths and transcript lengths and lower exon numbers, which agrees with previous studies [68]. The DELs involved in fat-related biological processes were identified between BH and BL. The low expression level and lack of annotation information of lncRNAs make it more challenging to explore lncRNA functions. To determine the regulatory mechanisms of lncRNAs, our study predicted the target genes of DELs by cis-regulation and trans-regulation. After the analysis of the DELs–target genes–KEGG pathway, our study identified many regulatory pathways related to fat metabolism and biosynthesis regulation. After combining the key genes located in those pathways and the DEGs, the candidate pathways associated with fat quality were identified. *ELOVL5*, targeted by TCONS_00261208, TCONS_00261206, TCONS_00054349, and TCONS_00252347, participated in the fatty acid metabolism pathway. *SCD*, targeted by TCONS_00173059 and TCONS_00138534, was enriched in the PPAR signaling pathway. *DGAT2*, targeted by TCONS_00282681, was involved in glycerolipid metabolism. *SLC24A5*, targeted by TCONS_00151368, TCONS_00014708, TCONS_00296593 and TCONS_00093273, and *TYRP1*, targeted by TCONS_00111522, TCONS_00281166, TCONS_00300482, TCONS_00304616, TCONS_00144414, TCONS_00140803, and TCONS_00252347, were strongly enriched in the biological process terms related to pigmentation. So *ELOVL5*, *SCD*, *DGAT2*, *SLC24A5,* and *TYRP1* candidate genes and the pathways in which they participate can be considered to play a key and important role in regulating the backfat quality of Beijing Black pigs. The candidate genes, related lncRNAs, and pathways involved in the regulation of backfat quality in Beijing Black pigs were obtained in this study. However, there is still much work to be done on pig backfat quality, especially to verify the real effects of these candidate genes and lncRNAs. The expression and modification of candidate proteins could be studied, and the molecular mechanism needs to be performed by testing on cells and even individual animals using molecular biological methods. 

## 5. Conclusions

In summary, this study’s insights are the first to clarify the expression profiles and function networks of mRNAs-lncRNAs in backfat tissues between diverging backfat qualities groups of Beijing Black pigs (BH and BL). The integrated analyses of DEGs and DELs obtained the candidate genes for strongly regulating backfat quality, such as *ELOVL5*, *SCD*, *DGAT2*, *SLC24A5,* and *TYRP1*. These findings provide a foundation for further studies and offer new insights elucidating the regulatory mechanisms of porcine backfat quality.

## Figures and Tables

**Table 1:** The phenotypic values of backfat quality of BH and BL groups.

Phenotypic Traits	BH	BL	P
Backfat colour/b value	4.52 ± 0.32	6.43 ± 0.50	4.8 × 10³ **
Backfat firmness/N	257.69 ± 45.49	123.82 ± 12.73	7.99 × 10³ **

** indicates significant differences between at *p* < 0.05.

## Data Availability

Data is contained within the article and supplementary materials.

## Supplementary Materials

The following supporting information can be downloaded at: https://www.mdpi.com/article/10.3390/foods11223654/s1; Table S1: The primers of genes and lncRNAs for qRT-PCR; Table S2: The statistics of raw and clean data after quality assessment. Table S3: Statistics of reference genome comparison results. Table S4: The differentially expressed mRNAs of backfat tissues between high backfat quality pigs and low backfat quality pigs. Table S5: The differentially expressed lncRNAs of backfat tissues between high backfat quality pigs and low backfat quality pigs. Table S6: The potential cis -targets of differentially expressed lncRNAs. Table S7: The potential trans-targets of differentially expressed lncRNAs. Figure S1: Genomic characteristics of mRNAs and lncRNAs in pig backfat.
