# Supplementary material for: Integrated Analysis of Long Non-Coding RNA and mRNA to Reveal Putative Candidate Genes Associated with Backfat Quality in Beijing Black Pig
Source: Foods. 2022 Nov 15;11(22):3654. doi: 10.3390/foods11223654 (PMC9689697; doi:10.3390/foods11223654)
Supplement: Supplementary file 1 [file foods-11-03654-s001.zip › Figure S1.pdf]

A

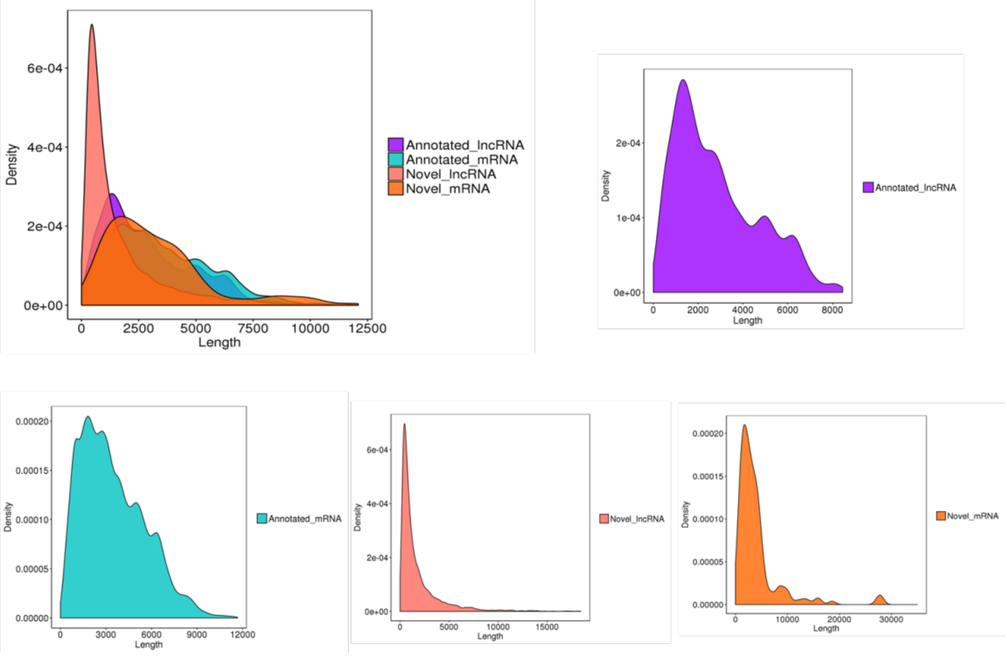
Density
Length
6e-04
4e-04
2e-04
0e+00
0
2500
5000
7500
10000
12500
Annotated_lncRNA
Annotated_mRNA
Novel_lncRNA
Novel_mRNA

B

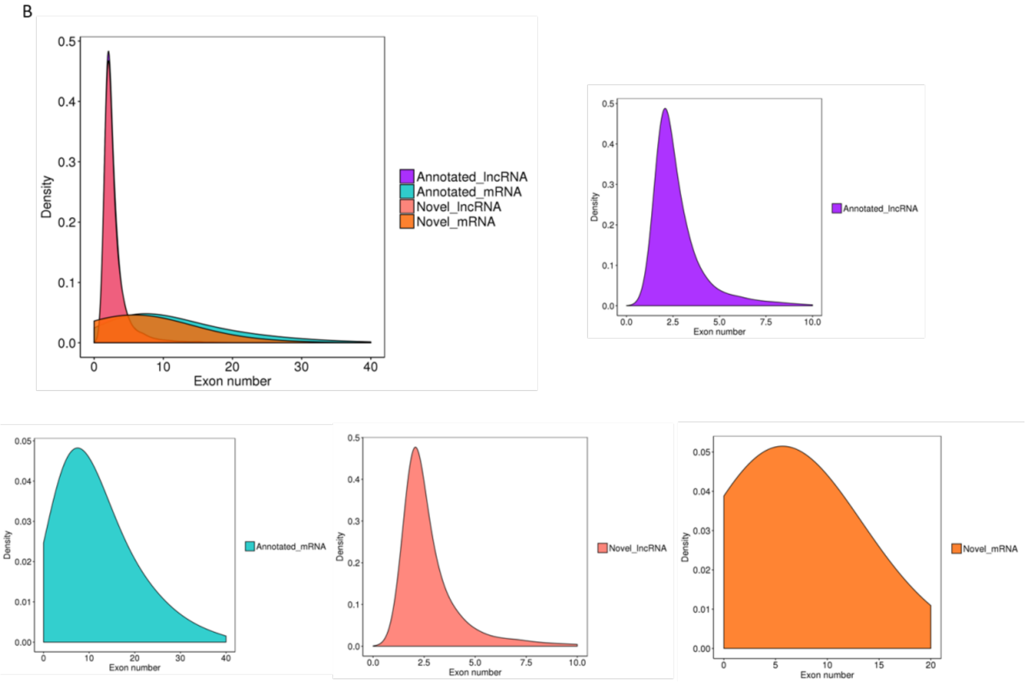
Density
Exon number
0.5
0.4
0.3
0.2
0.1
0.0
0
10
20
30
40
Annotated_lncRNA
Annotated_mRNA
Novel_lncRNA
Novel_mRNA

C

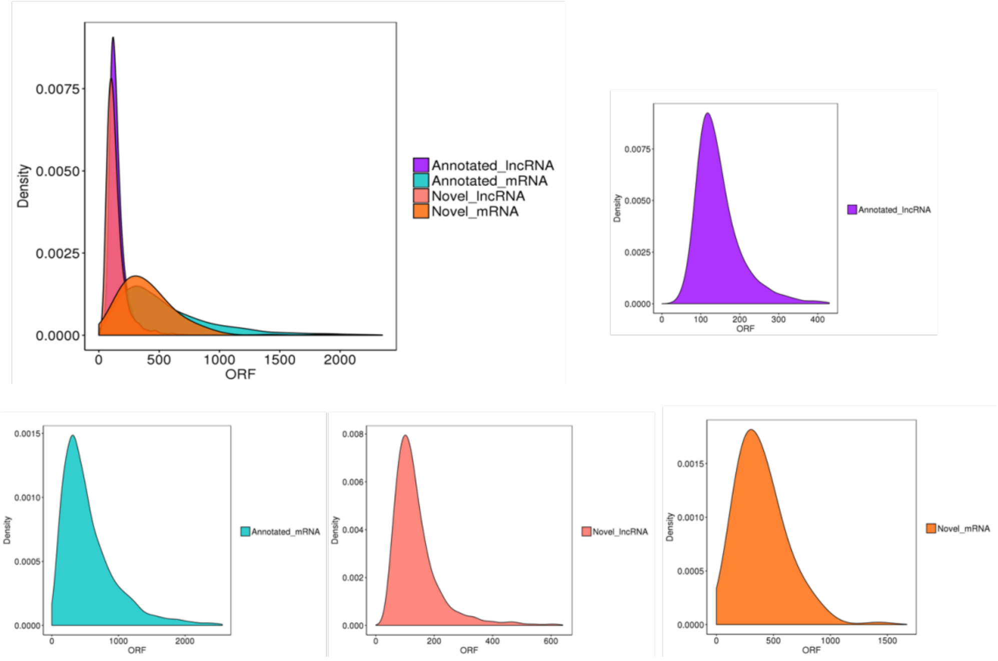

Figure S1. Genomic characteristics of mRNAs and lncRNAs in pig backfat (**A**) Length distribution of annotated mRNAs, annotated lncRNAs, novel mRNAs and novel lncRNAs; (**B**) Exon number distribution of annotated mRNAs, annotated lncRNAs, novel mRNAs and novel lncRNAs; (**C**) ORF length distribution of annotated mRNAs, annotated lncRNAs, novel mRNAs and novel lncRNAs.
